# TWEAK/Fn14 Axis: A Promising Target for the Treatment of Cardiovascular Diseases

**DOI:** 10.3389/fimmu.2014.00003

**Published:** 2014-01-20

**Authors:** Luis M. Blanco-Colio

**Affiliations:** ^1^Vascular Research Laboratory, IIS-Fundación Jiménez Díaz, Madrid, Spain

**Keywords:** atherosclerosis, stroke, biomarker, TWEAK, TNF superfamily

## Abstract

Cardiovascular diseases (CVD) are the first cause of mortality in Western countries. CVD include several pathologies such as coronary heart disease, stroke or cerebrovascular accident, congestive heart failure, peripheral arterial disease, and aortic aneurysm, among others. Interaction between members of the tumor necrosis factor (TNF) superfamily and their receptors elicits several biological actions that could participate in CVD. TNF-like weak inducer of apoptosis (TWEAK) and its functional receptor and fibroblast growth factor-inducible molecule 14 (Fn14) are two proteins belonging to the TNF superfamily that activate NF-κB by both canonical and non-canonical pathways and regulate several cell functions such as proliferation, migration, differentiation, cell death, inflammation, and angiogenesis. TWEAK/Fn14 axis plays a beneficial role in tissue repair after acute injury. However, persistent TWEAK/Fn14 activation mediated by blocking experiments or overexpression experiments in animal models has shown an important role of this axis in the pathological remodeling underlying CVD. In this review, we summarize the role of TWEAK/Fn14 pathway in the development of CVD, focusing on atherosclerosis and stroke and the molecular mechanisms by which TWEAK/Fn14 interaction participates in these pathologies. We also review the role of the soluble form of TWEAK as a biomarker for the diagnosis and prognosis of CVD. Finally, we highlight the results obtained with other members of the TNF superfamily that also activate canonical and non-canonical NF-κB pathway.

## Introduction

Cardiovascular diseases (CVD) claim more lives worldwide than any other, causing an estimated 17 million deaths worldwide each year due to heart attacks and strokes. CVD included several pathologies such as coronary heart disease, stroke or cerebrovascular accident, congestive heart failure, peripheral arterial disease, and aortic aneurysm, among others.

Cumulative evidence supports the important role of the tumor necrosis factor (TNF) superfamily of proteins in the development of CVD. The majority of ligands included in this family are synthesized as type II transmembrane proteins with a common structural motif (THD) that mediates self-trimerization and receptor binding (1). The extracellular domain of ligands can be cleaved to generate soluble cytokines. Receptors are usually type I transmembrane glycoproteins characterized by the presence of extracellular cysteine-rich domains (1). As their ligands, functional receptors are also usually trimerics. Many TNF members, including TNF-like weak inducer of apoptosis (TWEAK), activate the nuclear factor kappaB (NF-κB) family of transcription factors (2). NF-κB DNA-binding complex are homo- or heterodimers of five Rel proteins: NF-κB1 (p50), NF-κB2 (p52), RelA (p65), RelB, and c-Rel. NF-κB dimers translocate to the nucleus and bind to DNA by two different pathways, canonical or non-canonical NF-κB activation. Binding of NF-κB to DNA activates the transcription of several target genes that are implicated in the inflammatory response as well as in cell proliferation, migration, and differentiation. All these processes are closely related to pathological vascular remodeling.

## TWEAK and Fn14: Structure, Expression, and Function

The human TWEAK gene is located at chromosomal position 17p13.1 and encodes a 249-amino acid (aa) type II transmembrane protein (3). TWEAK is expressed as a full-length, membrane-bound protein (mTWEAK) and then is proteolytically processed by furin, leading to the release of a 156-aa, 18 kDa soluble form (sTWEAK) (3). The extracellular domain contains the receptor-binding site and the intracellular domain contains a putative serine phosphorylation site. In 2001, Fn14 was identified as the functional TWEAK receptor using a cDNA expression library screening approach (4). The human Fn14 gene is located at the chromosomal position 16p13.3 (5), and encodes a 129-aa type I transmembrane protein of 14 kDa that is processed into a mature form of 102-aa (4). The extracellular domain contains the ligand-binding site and the intracellular domain contains a TNFR-associated factor (TRAF)-binding site (6) implicated in signal transduction induced by TWEAK (7). A second receptor for TWEAK, CD163, has recently been identified (8, 9). CD163 is a hemoglobin scavenger receptor that is exclusively expressed by monocytes/macrophages (10). It has been proposed that CD163 acts as a scavenger receptor for TWEAK, thus preventing TWEAK from exerting its biological actions by sequestering it from the environment. However, it has been reported that recombinant CD163 failed to decrease cell death induced by TWEAK in macrophages (11). The relevance of TWEAK/CD163 interaction needs to be confirmed and more studies are needed in order to determine whether this interaction takes place either *in vitro* or *in vivo*. In addition, the existence of a third alternative receptor for TWEAK has been proposed, since murine RAW264.7 cells differentiation induced by TWEAK occurs in an Fn14-independent manner (12). However, no reports describing this alternative receptor have been published and we have observed that this monocytic/macrophage cell line expresses functional Fn14 (Blanco-Colio, unpublished observation).

TNF-like weak inducer of apoptosis is expressed in several cell types and tissues including the intestine, pancreas, lung, brain, ovary, skeletal muscle, and vasculature, and to a lesser degree in kidney and liver (3). Although TWEAK can be upregulated after injury (13), changes in TWEAK gene expression are usually moderated. By contrast, Fn14 expression in healthy tissues, including the vasculature and heart, is usually low or undetectable, although it is rapidly and highly upregulated under pathological conditions as demonstrated in experimental models of chronic liver injury (14), myocardial infarction (15), colitis (16), denervation-induced skeletal muscle atrophy (17), restenosis after balloon injury (4), atherosclerosis (18), autoimmune encephalomyelitis (19), acute kidney injury (20), and cardiac dysfunction (21). Once Fn14 is upregulated, TWEAK binds and causes Fn14 trimerization and signal transduction (7). Although soluble TWEAK is responsible for the responses associated with Fn14, it has been recently reported that full-length, membrane-anchored TWEAK can, in a juxtacrine manner, bind to Fn14 on neighboring cells and activate the NF-κB signaling pathway, thus initiating the cellular response (22).

**Figure 1:**
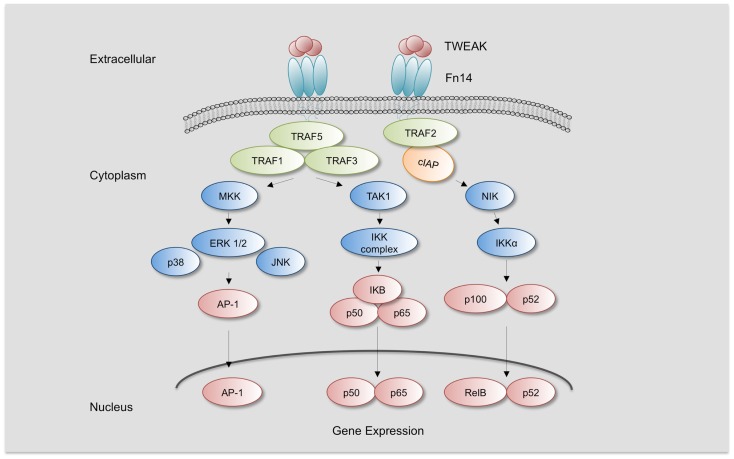
**TWEAK/Fn14 Signaling**. TWEAK/Fn14 binding induces the recruitment of cIAP and TRAF-1, 2, 3, and 5 and leads to activation of different kinases such as mitogen-activated protein kinase kinases (MKK), transforming growth factor β-activated kinase 1 (TAK1), and NF-κB inducing kinase (NIK). TAK1 activates IKKβ and NIK activates IKKα, leading to the activation of canonical or non-canonical NF-κB pathway, respectively. MMK are responsible for the activation of c-Jun N-terminal kinase 1 (JNK1) and p38 MAPK, which activate transcription factor activator protein-1 (AP-1). Increased activation of NF-κB and AP-1 leads to the expression of specific target genes responsible for TWEAK-mediated responses.

Fn14 is upregulated by several growth factors, cytokines, and interleukins in cells present in the injured vascular wall such as endothelial cells, vascular smooth muscle cells (SMCs), and monocyte/macrophages, but not in T and B lymphocytes (4, 18, 23, 24). However, little is known of the regulatory mechanisms of Fn14 expression, and only the RhoA/ROCK pathway has been related to Fn14 upregulation in cardiomyocytes (25). TWEAK protein can be upregulated by PMA and IFN-γ in cultured peripheral mononuclear cells and natural killer cells (24, 26). Fn14 trimerization induces the recruitment of TRAF2 and TRAF5 through its TRAF-binding motif (PIEET). This motif is responsible for activating different signaling pathways such as NF-κB and mitogen-activated protein kinases (MAPK) (Figure 1) (7, 27). Activation of NF-κB by TWEAK participates in the upregulation of several cytokines implicated in the recruitment of inflammatory cells within the injured vessel wall. Thus, TWEAK increases MCP-1 and RANTES in SMCs (28). TWEAK also activates NF-κB in cultured Thp-1 monocytic cell line (29). In addition, TWEAK induces the expression of CCL19 and CCL21 in murine tubular cells (30), and both cytokines are also expressed in atherosclerotic plaques of ApoE-deficient mice, a model of hyperlipidemic-induced atherosclerosis (31). TWEAK also activates MAPK, although activation of ERK, c-Jun N-terminal kinase (JNK), or p38 pathways is context-dependent. MAPK activation has been reported in several cell lines, including Thp-1 monocytic cell line, endothelial cells, cardiomyocytes, fibroblast, and others (23, 29, 32–34). There are also different reports indicating that TWEAK activates PI3K/AKT in different cell types. Thus, TWEAK increases HMGB1 secretion by cultured monocytes through PI3K activation (29). In addition, TWEAK also activates transforming growth factor-β activated kinase 1 (TAK1), implicated in NF-κB activation (35, 36) and JNK, related with AP-1 activation (37). Overall, TWEAK activates several signaling pathways that participate in the inflammatory response of the injured tissues.

**Figure 2:**
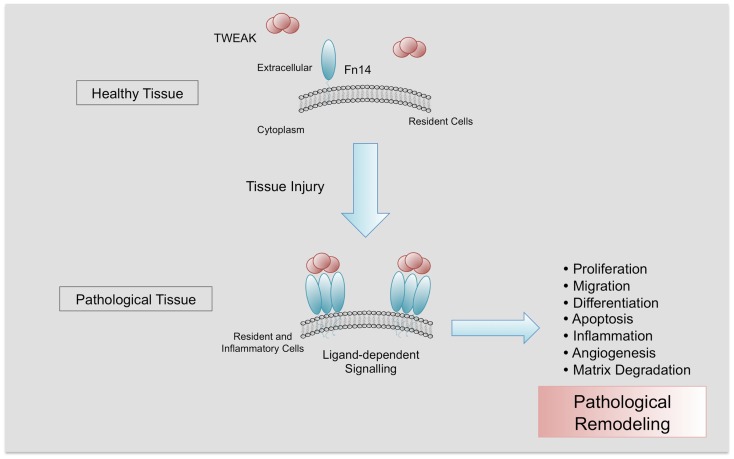
**Pathological actions of TWEAK/Fn14 interaction**. Fn14 is almost absent in healthy tissues. After injury, Fn14 is upregulated, facilitating the interaction with its ligand TWEAK and its trimerization. In a context of chronic injury, TWEAK/Fn14 interaction participates in pathological tissue remodeling, promoting proliferation, migration, differentiation, apoptosis, inflammation, angiogenesis, and matrix degradation.

Several functions with potential pathological significance have been related to TWEAK/Fn14 interaction and are dependent on the cell type, microenvironment, and cell activation. However, the basis for these differential responses is poorly understood. TWEAK can regulate cell proliferation, migration, differentiation, and death as well as tissue inflammation, angiogenesis, and regeneration (Figure 2) (3, 5, 38–40). The precise role of TWEAK in different pathological situations needs to be characterized, since TWEAK has beneficial or deleterious effects depending on the stage of the disease (13, 41).

## TWEAK and Atherosclerosis

**Figure 3:**
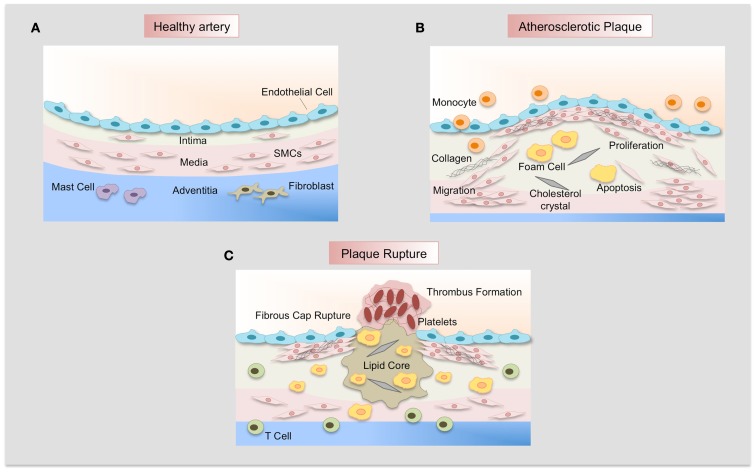
**Atherosclerotic plaque development**. **(A)** Normal artery is formed by a monolayer of endothelial cells, the tunica media of resident smooth muscle cells embedded in an extracellular matrix, and the adventitia that contains mast cells, fibroblasts, and microvessels. **(B)** Adhesion of leukocytes to dysfunctional endothelium leads to their migration into the vascular wall, forming the neointima. Monocytes differentiate to macrophages and uptake lipids yielding foam cells. SMCs migrate from the media, proliferate, and synthesize extracellular matrix proteins such as collagen and elastin. SMCs and macrophages can die and lipid derived from dead cells accumulates in the central region of the plaque, the necrotic core that is covered by a fibrous cap. **(C)** Finally, when the fibrous cap is broken, blood coagulant components trigger thrombus formation, which occludes the lumen and interrupts the blood flow.

Atherosclerosis is a multifactorial disease characterized by chronic inflammation and excessive cell proliferation (Figure 3). Vascular lesions begin as a fatty streak in the subendothelial space of large arteries. Endothelial cells acquire an activated phenotype and express adhesion molecules such as intercellular adhesion molecules (ICAMs), selectins, and vascular adhesion molecules (VCAMs) that act as receptors for proteins expressed by leukocytes (monocytes and lymphocytes and neutrophils). Recruitment of monocytes to the subendothelial space causes their differentiation to macrophages that uptake oxidized low density lipoproteins (ox-LDL). In addition, chemokines and cytokines are secreted by inflammatory cells and induce proliferation and migration of SMCs from the media forming the neointima (42). The transition of relatively early lesions to more advanced lesions is characterized by the proliferation of SMCs and continuous uptake of ox-LDL by macrophages, forming foam cells. In addition, SMCs synthesize extracellular matrix proteins that lead to the development of the fibrous cap. This cap confers resistance to rupture by the accumulation of collagen synthesized by SMCs. The continuous ingestion of ox-LDL by foam cells induces death of these cells, releasing of insoluble lipids and contributing to the formation of the necrotic core characteristic of advanced lesions. Expression of different proteases by macrophages and SMCs leads to degradation of the fibrous cap, promoting plaque instability and subsequent plaque rupture. Rupture of an atherosclerotic plaque may result in the occlusion of an artery by the formation of a thrombus over an atherosclerotic lesion, causing myocardial infarction, stroke, or peripheral vascular disease (42).

The TWEAK/Fn14 axis plays an important role in several steps of atherosclerotic plaque development including initiation, progression, destabilization/rupture, and subsequent thrombosis. As commented, TWEAK is expressed in both the normal and pathological arterial wall (18), but Fn14 is almost absent in healthy arteries and its expression is highly upregulated in the carotid artery (18), femoral atherosclerotic plaques (43), and in abdominal aortic aneurysms (44). Different stimuli induce Fn14 expression in resident and inflammatory cells present in the vascular wall. Thus, pro-inflammatory cytokines (IL-1β and INF-γ), growth factors (PDGF-BB, EGF, FGF-2), Angiotensin II, or α-thrombin increase Fn14 expression in human and rat aortic SMCs (4, 18). In addition, VEGF-A and FGF-2 increase Fn14 expression in human umbilical endothelial cells (23) and human CD14^+^ monocytes express Fn14 in response to INF-γ or PMA stimulation (24).

In the first steps of atherosclerosis development, adhesion molecules and chemokines are responsible for the recruitment of the inflammatory cells to the injured tissue. *In vitro* studies have demonstrated that TWEAK/Fn14 interaction induces expression of adhesion molecules such as ICAM-1 and E-selectin in human umbilical endothelial cells (45). Furthermore, TWEAK increases interleukin-8 and MCP-1 secretion by endothelial cells (45). Infiltrating cells secrete many cytokines that contribute to SMCs migration and proliferation, favoring plaque progression. In this sense, TWEAK/Fn14 interaction directly induces proliferation and migration of human and rat aortic SMCs (39, 45, 46) and human endothelial cells (4, 23, 39, 45). These suggest that the TWEAK/Fn14 axis could participate in neointimal thickening of the pathological arterial wall. In fact, an *in vivo* study has reported that Fn14 is upregulated in SMCs after balloon injury in mice (4).

The presence of a chronic inflammatory response is an important phenomenon implicated in the development and progression of atherosclerotic plaque. A key transcription factor implicated in vascular inflammation is NF-κB. Activation of signal transduction mediated by NF-κB has been demonstrated at different stages of atherosclerotic lesion development, from plaque formation to plaque rupture (47). NF-κB is activated in SMCs, macrophages, and endothelial cells in human atherosclerotic plaques (48–50). Several molecules can activate this transcription factor in the context of atherogenesis. Inflammatory stimuli such as members of the TNF-α superfamily, IL-1, and ox-LDL induce NF-κB activation, and in consequence amplifying and maintaining a vascular inflammatory response that facilitates atherosclerosis progression. Activation of this transcription factor in endothelial cells enhances the expression of adhesion molecules, chemokines, and metalloproteinases (MMP). These molecules coordinate the invasion of inflammatory cells into the vascular wall, and enhance migration and proliferation of SMCs as well as the remodeling of the extracellular matrix. Inflammatory cells and SMCs also increase cytokine and MMP expression through NF-κB activation, perpetuating the inflammatory response. In particular, TWEAK activates NF-κB in several cell types and increases the expression of pro-inflammatory proteins such as IL-6, IL-8, MCP-1, and RANTES (7, 18, 27, 28, 46, 51, 52); these pro-inflammatory proteins are implicated in atherogenesis. In addition, recombinant TWEAK injection increases atherosclerotic lesion size and inflammatory cell content as well as NF-κB activation in the aortic root of hyperlipidemic ApoE-knockout mice (28). Moreover, anti-TWEAK monoclonal antibody (mAb) therapy diminishes NF-κB activation as well as inflammatory response in ApoE-null mice, indicating that endogenous TWEAK participates in atherogenesis (28). In addition, genetic deletion of TWEAK or treatment with anti-TWEAK mAb diminished NF-κB activation, chemokines secretion and inflammatory response in ApoE-deficient mice (53). The activation of NF-κB by TWEAK observed in this experimental model was related to the canonical pathway, since p50/p65 dimers were detected in the nuclei of cells within atherosclerotic plaques. Until now, non-canonical NF-κB activation induced by TWEAK has not been reported in atherosclerotic plaques.

TWEAK also increases the secretion of HMGB1 through NF-κB activation in human M1 macrophages (29). HMGB1 is a DNA-binding cytokine that activates endothelial cells and monocytes/macrophages to express pro-inflammatory cytokines, chemokines, and adhesion molecules functioning as a critical mediator of inflammation (54). HMGB1 colocalizes with Fn14 in the shoulder region of human atherosclerotic plaques, a macrophage-rich area (29). In addition, systemic injection of recombinant TWEAK augmented HMGB1 expression in atherosclerotic plaques of hyperlipidemic ApoE-null mice (29). The importance of the finding that NF-κB can regulate HMGB1 release induced by TWEAK is because secreted HMGB1 may in turn induce NF-κB activation, forming a loop between NF-κB and HMGB1 that perpetuates vascular pro-inflammatory effects related to TWEAK. These data support the notion that TWEAK/Fn14 interaction has deleterious consequences in the injured vascular wall.

Interestingly, it has been reported that TWEAK can modulate macrophage size within atherosclerotic plaques (55). This finding is related to the capacity of TWEAK to modulate lipid uptake by macrophages. In fact, ApoE-deficient mice treated with Fn14–Fc protein present smaller macrophages in their atherosclerotic plaques, and treatment with anti-Fn14 or anti-TWEAK antibodies or Fn14–Fc protein diminished macrophage uptake of modified lipids *in vitro* (55).

The stability of the advanced atherosclerotic plaque depends on the integrity of the fibrous cap that encloses its lipid core. Established atherosclerotic lesions usually have a dense fibrous cap. However, areas with sustained inflammation, macrophage accumulation, and apoptosis are prone to rupture due to a weakening of the fibrous cap. Deterioration of the fibrous cup is dependent on the activity of MMP, which are collagen-degrading endopeptidases that are secreted by SMCs and macrophages (56). As commented, TWEAK and Fn14 are expressed in macrophages/foam cells rich regions of atheroma plaques and colocalized with MMP (57). Moreover, an activating anti-Fn14 antibody increases the expression of MMP-9 and MMP-1/13 in cultured monocytes (57). In addition, anti-TWEAK mAb treatment diminishes MMP activity in atherosclerotic plaques present in the aortic root of ApoE-deficient mice (53). In addition, features of greater plaque stability included augmented collagen/lipid ratio, reduced macrophages content, and less presence of lateral xanthomas, buried caps, medial erosion, intraplaque hemorrhage, and calcium content have been observed in TWEAK/ApoE-double knockout mice or in anti-TWEAK mAb treated ApoE-deficient mice (53). These data indicate a potential role of TWEAK in extracellular matrix degradation, which favors plaque instability.

Plaque rupture or erosion, and subsequent thrombosis, represent the main complications of atherosclerosis and could lead to an acute cardiovascular event. Different molecules, such as plasminogen activator inhibitor 1 (PAI-1) and tissue factor (TF), are responsible for hemostasis and thrombosis (58). TF is the principal initiator of the clotting cascade, while PAI-1 plays a critical role in inhibiting fibrinolysis, and thereby the activity of both molecules promotes thrombotic states and plays a crucial role in vascular diseases (59). Fn14 colocalizes with PAI-1 and TF in human carotid atherosclerotic plaques (60). In addition, TWEAK increases TF and PAI-1 mRNA and protein expression as well as activity in cultured human aortic SMCs (60). Furthermore, systemic injection of recombinant TWEAK augmented TF and PAI-1 expression in atherosclerotic plaques of ApoE-deficient mice and, conversely, anti-TWEAK treatment diminished the expression of both prothrombotic proteins (60). These data indicate that TWEAK favors thrombus formation after plaque rupture.

Overall, data obtained from *in vitro* and *in vivo* studies indicate that TWEAK participates in different stages of atherosclerotic plaque development from early stages to progression and subsequent plaque rupture that lead to an acute cardiovascular event, such as myocardial infarction or stroke. Anti-TWEAK treatment has the capacity to diminish pro-inflammatory response associated with atherosclerotic plaque progression and to alter plaque morphology toward a stable phenotype.

## TWEAK and Stroke Outcome

Stroke is the third most common cause of death in the world (61). A stroke, or cerebrovascular accident, causes rapid loss of brain function due to a lack of oxygen; sudden death of brain cells also takes place. The two main types of stroke include ischemic (when a blood clot or thrombus forms) and hemorrhagic stroke. The outcome after a stroke depends on where it occurs and how much of the brain is affected. Smaller strokes may result in minor problems, such as weakness in an arm or leg. Larger strokes may lead to paralysis or death. Ischemic stroke triggers a cascade of pathophysiological events such as energy depletion, excitotoxicity, peri-infarct depolarization, inflammation, and apoptotic cell death (62). The onset of the ischemic insult is followed by an increase in the expression of pro-inflammatory molecules in the ischemic tissue, which has been associated with neuronal death and poor outcome.

Recent reports have shown the role of TWEAK/Fn14 axis after an ischemic stroke. In fact, it has been reported that ischemic stroke in humans (63) and experimental middle cerebral artery occlusion (MCAO) in mice (64, 65) increase the expression of both TWEAK and Fn14 in the ischemic tissue. In the central nervous system (CNS), TWEAK and Fn14 are expressed mainly in endothelial cells, perivascular astrocytes, microglia, and neurons. There are two principal mechanisms by which TWEAK participates in stroke pathogenesis: neuronal apoptosis and breakdown of the blood–brain barrier (BBB) (64, 66). It has been demonstrated that in response to hypoxia/ischemia, TWEAK induces cell death in neurons via NF-κB activation and PARP-1 and caspase-3 cleavage (64, 67). However, oxygen–glucose deprivation conditions did not affect cell survival in neurons from Fn14- or TWEAK-deficient mice, indicating that cell death is mediated by the TWEAK/Fn14 interaction (67).

On the other hand, during cerebral ischemia, disruption of the architecture of the neurovascular unit (NVU) results in an increase in the permeability of the BBB with the development of cerebral edema, which is a major cause of mortality among patients with acute stroke. NVU is a dynamic structure consisting of endothelial cells, the basal lamina, astrocytic end-feet processes, pericytes, and neurons (68). The permeability of BBB is increased by pro-inflammatory cytokines that act on the NVU under ischemic conditions (69). It has been demonstrated that TWEAK has a detrimental effect on the structure of the NVU and the permeability of the BBB in the early stages of cerebral ischemia. Recombinant TWEAK injection directly into the brain induces activation of NF-κB and MMP-9 expression, resulting in the disruption of the structure of NVU and an increase in the permeability of BBB (70). Furthermore, inhibition of TWEAK actions by Fn14–Fc decoy receptor or Fn14 deficiency diminished cerebral ischemia-induced increase in the permeability of the NVU. This protection was associated with a faster recovery of locomotor activity (66).

Finally, intraperitoneal administration of anti-TWEAK monoclonal antibodies (64) or intracerebroventricular administration of Fn14–Fc decoy receptor (65) diminished the infarct size by around 30–40% after 48 h of MCAO. In addition, Fn14-deficient mice exhibited a 60% reduction in the volume of the ischemic lesion following MCAO compared to wild-type animals (66). Overall, TWEAK may play an important role during ischemia-induced brain injury and its inhibition in the brain could be a novel neuroprotective strategy for the treatment of ischemic stroke.

## Diagnostic and Prognostic Value of Soluble TWEAK for Cardiovascular Diseases

**Figure 4:**
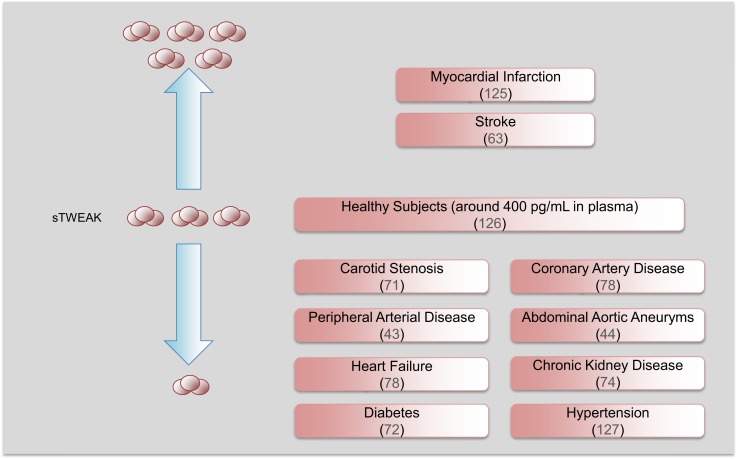
**Soluble TWEAK as a biomarker of cardiovascular diseases**. Circulating sTWEAK concentrations are diminished in subjects with chronic vascular damage including carotid stenosis, coronary artery disease, peripheral artery disease, abdominal aortic aneurysm, heart failure, and chronic kidney disease. Soluble TWEAK levels are also diminished in subjects with cardiovascular risk factors such as diabetes or hypertension. However, circulating sTWEAK levels are increased in patients suffering an acute cardiovascular event (myocardial infarction or stroke).

As commented above, TWEAK is expressed as a full-length, membrane-bound protein and then is proteolytically processed by furin, leading to the release of a 156-amino acid, 18 kDa soluble form (sTWEAK) (3). Among the potential biomarkers that could be differentially secreted by the pathological arterial wall, sTWEAK was identified as a protein that is highly released by normal arteries in comparison with carotid atherosclerotic plaques (71). Plasma levels of sTWEAK were also found to be diminished in patients with atherosclerosis compared to control subjects. The association of sTWEAK with the presence of CVD or CVD-related diseases has been extensively validated in other cohorts of individuals (Figure 4). Thus, sTWEAK concentrations were significantly reduced in patients with chronic kidney disease (CKD) and/or type II diabetes (72). In addition, sTWEAK plasma concentrations were diminished in patients undergoing hemodialysis compared to healthy subjects (73). In addition, a gradual decrease in sTWEAK along with a reduction in estimated glomerular filtration rate in CKD patients has been observed (74–76). Reduced levels of sTWEAK have also been associated with the presence of coronary artery disease (CAD) (25, 77), systolic heart failure (78), peripheral artery disease (PAD) (43), and aortic abdominal aneurysm (AAA) (44). Finally, elevated circulating sTWEAK levels have been described in patients after myocardial infarction (25) and stroke (63). However, although the study of circulating proteins in subjects suffering an acute event could unveil novel proteins implicated in atherothrombosis, some of these proteins could be released by the tissue necrosis that takes place during an acute event.

Two different surrogate markers of atherosclerosis such as flow-mediated dilation (FMD) and intima/media thickness (IMT) have been negatively associated with sTWEAK concentrations (71, 74, 75). As mentioned previously, endothelial dysfunction participates in the formation of vascular lesion (79). FMD described the vasodilation of a conduit artery following an augmentation in shear stress induced by an ischemia period. FMD provides us information about the integrity of the endothelium (80). It has been reported that sTWEAK plasma concentrations are negatively associated with FMD in patients with CKD (74), persisting after adjustment for factors related to FMD in CKD subjects such as blood pressure, C-reactive protein, and estimated glomerular filtration rate. This result was later confirmed in hypertensive CKD patients (75). In addition, IMT can be measured non-invasively by means of B-mode ultrasound, and increases in IMT have been associated with an augmentation for future cardiovascular outcomes (81). Thus, IMT has been negatively associated with sTWEAK concentrations in asymptomatic subjects (71) and in patients with CKD (76, 82, 83), even after adjustment for traditional risk factors and inflammatory biomarkers. Moreover, sTWEAK is associated with atherosclerotic burden in CKD patients (83). However, IMT was positively correlated with sTWEAK in renal transplant patients (84, 85). Overall, the association of sTWEAK levels with different surrogate markers of atherosclerosis indicates that this protein could be a novel and independent biomarker of CVD.

Finally, different reports have indicated the potential use of sTWEAK as a prognostic biomarker of CVD or CVD-related diseases as well as its impact on survival. Thus, individuals in the upper two tertiles of sTWEAK concentrations presented a lower incidence of PAD (43). In addition, a decreased sTWEAK concentration was significantly and independently associated with long-term cardiovascular mortality in patients with lower-extremity PAD (86). sTWEAK levels were also negatively related with AAA size and AAA expansion rate after a 5-year follow-up, and sTWEAK concentrations were predictive for subjects expanding more than 2 mm/year in AAA size (44). As for CKD, decreasing sTWEAK concentration was associated with increased risk of cardiovascular events independently of basic confounders (age, gender, estimated glomerular filtration rate, C-reactive protein, diabetes, and cardiovascular comorbidity) (76). However, high levels of sTWEAK were associated with atherosclerosis in patients with systemic lupus erythematosus (SLE), but not in control subjects (87). In addition, although sTWEAK plasma levels were diminished in HD patients compared with controls, subjects belonging to the upper tertile of sTWEAK presented a higher risk of all-cause and cardiovascular mortality (73). This discrepancy could be due to the existence of the known reverse epidemiology observed in HD patients. Finally, sTWEAK also provides prognostic information on subjects with heart failure. Subjects with chronic stable heart failure with reduced sTWEAK plasma concentrations (78) present a higher mortality rate than those with elevated sTWEAK levels. In addition, the increase of sTWEAK concentrations diminishes the risk of mortality in subjects with non-ischemic heart failure (88).

**Figure 5:**
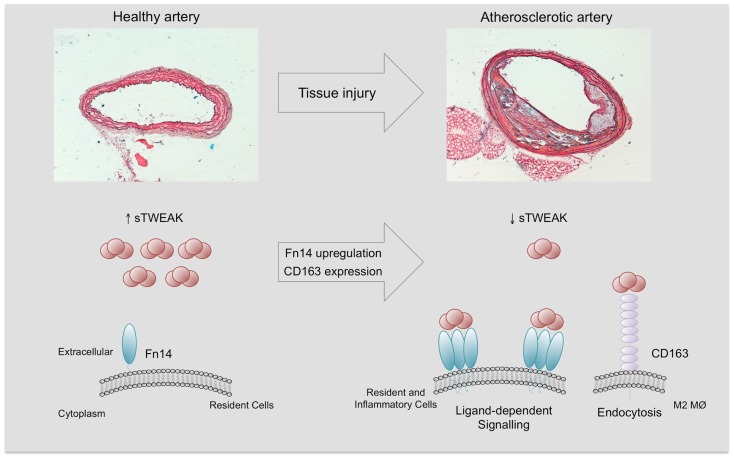
**Soluble TWEAK in health and disease**. Fn14 expression is almost absent and minimal Fn14 activation is expected in the healthy arterial wall. However, under pathological conditions, Fn14 expression is highly upregulated in resident (smooth muscle cells) and inflammatory cells (e.g., M1 macrophages). This increase facilitates the interaction with sTWEAK and would trigger sTWEAK-dependent Fn14 signaling. On the other hand, pathological tissues are infiltrated by anti- inflammatory macrophages (M2) that express CD163, a scavenger receptor of sTWEAK. The tissue consumption of sTWEAK by Fn14 interaction and CD163 degradation could be responsible for the decrease in circulating sTWEAK levels observed in subjects with atherosclerostic complications.

The mechanism/s by which sTWEAK is diminished in subjects with vascular damage should be related to the expression of their receptors. As commented, Fn14 expression is undetectable in the vasculature in normal conditions (18). However, under pathological conditions including systemic inflammatory states, Fn14 is highly upregulated in the vasculature, favoring sTWEAK binding and retention in the pathological tissues (18, 57). In addition, the expression of CD163 by M2 macrophages in pathological tissues could be responsible for the decrease in sTWEAK, since CD163 can bind and internalize sTWEAK *in vitro* (8). On the basis of this preceding literature, we speculate that the reduction in sTWEAK concentrations in cardiovascular-related diseases could potentially reflect either Fn14 binding or CD163 degradation (Figure 5). However, this hypothesis needs to be tested in future studies. Overall, all these data reveal that sTWEAK could be a novel biomarker of CVD. More large-scale studies to consolidate its usefulness are required.

## Other TNF Superfamily Members Implicated in Atherothrombosis That Activate Both Canonical and Non-Canonical NF-κB Pathway

In recent years, the number of TNF receptors that are known to potentially activate the non-canonical NF-κB pathway has increased. These include, in addition to Fn14, CD40, B-cell activating factor receptor (BAFFR), lymphotoxin β receptor (LTβR), receptor activator of NF-κB (RANK), and CD27 (89–93). Some of these receptors have been implicated in different functions related to the pathogenesis of atherosclerosis (Table 1).

**Table 1:** **Effect of TNF superfamily members that activate non-canonical NF-κB in experimental models of murine atherosclerosis**.

TNF member	Experimental approach/animal model	Plaque size	Reference
TWEAK	Systemic rTWEAK injection in ApoE ^−/−^mice	Increased	Muñoz-García et al. (28 )
	Blocking TWEAK in ApoE^−/−^mice	Reduced	Muñoz-García et al. (28 )
Fn14	Blocking Fn14-Fc in ApoE ^−/−^mice	Reduced	Schapira et al. (55 )
CD40	CD40^−/−^/ApoE^−/−^double deficient mice	Reduced	Lutgens et al. (94)
	CD40^−/−^/LDLR^−/−^double deficient mice	No change	Zirlik et al. (95)
	CD40-TRAF6 blocking interaction in ApoE ^−/−^mice	Reduced	Lutgens et al. (94 )
CD40L	CD40L^−/−^/ApoE^−/−^double deficient mice	Reduced	Lutgens et al. (96)
	Blocking CD40L in ApoE^−/−^mice	No effect	Lutgens et al. (97 )
	Blocking CD40L in LDLR^−/−^mice	Reduced	Mach et al. (98 ), Schonbeck et al. (99 )
	BMT: CD40L^−/−^to LDLR^−/−^mice	No change	Bavendiek et al. (100), Smook et al. (101)
	Transfer CD40L^−/−^platelets into ApoE^−/−^mice	Reduced	Lievens et al. (102)
BAFFR	BAFFR^−/−^/ApoE^−/−^double deficient mice	Reduced	Kyaw et al. (103)
	BMT: BAFFR^−/−^to LDLR^−/−^mice	Reduced	Sage et al. (104)
	Blocking BAFFR antibody in ApoE^−/−^mice	Reduced	Kyaw et al. (105 )
LTβR	Blocking LTβR in ApoE^−/−^mice	No change	Gräbner et al. (106)
RANK	OPG^−/−^/ApoE^−/−^double deficient mice	Increased	Bennett et al. (107)
	Blocking Fc-OPG in ApoE^−/−^mice	No change	Morony et al. (108 )
CD70	CD70 tg/ApoE*3 Leiden mice	Reduced	van Olffen et al. (109 )

*BMT, bone-marrow transplantation; OPG, osteoprotegerin*.

The role of *CD40* and its ligand CD40L in atherosclerosis has been extensively studied. Both proteins are expressed in various cell types implicated in atherogenesis such as platelets, endothelial cells, monocytes/macrophages, and SMCs (110). CD40L induces a broad inflammatory response in these cell types, including increased expression of adhesion molecules, pro-inflammatory cytokines, matrix degrading enzymes, and pro-coagulants (111, 112). Different groups have analyzed the role of CD40 and CD40L in athero-prone mice by using diverse strategies such as gene modification, blocking antibody treatment, or bone-marrow transplantation (BMT). Thus, CD40L and ApoE double deficient mice develop markedly reduced atherosclerosis (96, 98, 100). In addition, treatment with neutralizing anti-CD40L antibodies diminished atherosclerotic lesion size (98, 99) in LDLR-deficient mice but failed to modify plaque size in ApoE-null mice (97). The decrease of atherosclerotic plaques was associated with features of higher plaque stability such as reduced macrophage and lipid content as well as increased collagen deposition. The effect of CD40L on atherosclerotic plaque progression seems to be related with resident cells (endothelial and SMCs), since BMT failed to modify atherosclerosis plaque size in LDLR^−/−^ mice (100, 101). However, a recent study describes that transfer of CD40L^−/−^ platelets into ApoE-null mice diminished atherosclerotic burden, an effect that results from the capacity of platelets to synthesize higher amounts of CD40L (102). The role of CD40 in atherosclerosis remains controversial. It has been demonstrated that CD40 and ApoE double deficient mice develop reduced levels of atherosclerosis when given a normal chow diet compared with control animals (94). However, a similar atherosclerotic burden was observed in CD40^−/−^LDLR^−/−^ mice on a high-cholesterol diet (95). These data could suggest that CD40L mediates atherosclerosis development independently of CD40 (95). However, specific interruption of CD40/TRAF6 interaction in ApoE-deficient mice diminished atherosclerotic plaque size, indicating that CD40L/CD40 interaction participates in atherosclerotic plaque development (94).

It has been demonstrated that the depletion of B cells diminished atherosclerosis in mice (113). The proatherogenic effect of B cells is mainly driven by the B2 subset, which responds to T-cell-dependent antigens and is part of the adaptive immune response (114), while the atheroprotective effect is attributed to the B1 subset, which responds to T-cell-independent antigens (115). The survival and maturation of B2 lymphocytes depends on the interaction of BAFF with its receptor, *BAFFR* (116). Genetic disruption of BAFFR induces a significant reduction in mature B2 cells without affecting B1a cells (117) and BAFFR/ApoE double deficient mice present a reduced atherosclerotic plaque size and macrophage content in their aortic root; this effect is also related to a decrease in the number of B2 cells (103). In addition, BMT of BAFFR deficient cells to LDLR^−/−^ mice also leads to a reduction in plaque size and inflammation (104). These data could suggest that BAFFR is an interesting therapeutic target to limit the development of atherosclerosis. Indeed, atherosclerosis development is diminished in ApoE^−/−^ treated with a BAFFR blocking mAB (105).

Until now, the role of *LTβR* in atherosclerotic plaque development is unclear. As commented above, cells present in atherosclerotic lesions elicit persistent inflammation and trigger immune adaptive response toward arterial wall-derived autoantigens, such as ox-LDL or heat shock proteins. Coronary patients with atherosclerosis present infiltrates of leukocytes in the adventitia, and the presence of adventitial B-cell follicle-like aggregates in human aorta has been demonstrated (118). Moreover, adventitia of ApoE-deficient mice also contains T- and B-cell aggregates. These aggregates are the precursors of fully structured aorta tertiary lymphoid organs (ATLOs) that contain a high number of germinal centers, endothelial venules, regulatory T cells, and LN-like conduits that connect ATLOs to medial SMCs (106). The recruitment of T- and B-cell aggregates is dependent on CXCL13 and CCL21 secretion by SMCs through an LTβR-dependent signaling pathway. The formation of ATLOs in ApoE-deficient mice is restricted to abdominal aorta, and ATLOs are communicated with medial SMC by conducts that serve as channels for both transport of molecules (e.g., cytokines and chemokines) and soluble antigens (119). Although it is conceivable that ATLOs play a role in atherosclerotic plaque progression, ApoE-null mice treated with anti-LTβR have not modified their atherosclerotic plaques. Future gene deletion studies would help to understand the role of LTβR and ATLOs in atherogenesis.

*RANKL,* which is expressed in human atherosclerotic plaques (120), is capable of modulating different cell-type activities through its receptor, RANK. RANK/RANKL interaction activates several intracellular signal transduction pathways such as MAPKs and NF-κB (121). Several proatherogenic actions of RANKL have been described. For example, RANKL induces MCP-1 expression and secretion and matrix MMP activity in SMCs (122). In addition, RANKL induces TF expression in macrophages mainly through the cooperative action of NF-κB, AP-1, and Egr-1, supporting a role of RANKL in the thrombogenicity of atherosclerotic plaques (123). Furthermore, RANK, RANKL, and the decoy receptor for RANKL osteoprotegerin (OPG) have been related with vascular calcification, a crucial step for plaque destabilization and rupture. In the absence of OPG, mice display vascular calcification and present increased atherosclerotic plaque size (107). However, treatment of LDLR-deficient mice with Fc-OPG diminished vascular calcification but failed to reduce atherosclerotic plaque size (108).

Finally, *CD27* is mainly found in T cells, and its ligand CD70 is expressed in B cells, activated T cells, and dendritic cells. Interaction of both proteins is necessary for the generation and long-term maintenance of T-cell immune responses (124). Overexpression of CD70 in B cells leads to an expansion of Th1 T cells in mice. This indicates that these mice should have a proatherogenic phenotype. However, mice overexpressing CD70 are protective against atherosclerosis development, possibly due to a reduced viability of circulating monocytes (109). After these results, further research is clearly needed to clarify the relevance of CD27 in atherosclerosis.

## Concluding Remarks

The evidence gathered to date supports a role of the TWEAK/Fn14 axis in the development and outcome of atherosclerosis and ischemic stroke. Data from experimental models make TWEAK and its functional receptor Fn14 a promising target for the treatment of patients with different CVD. Treatment with the TWEAK neutralizing antibody or Fn14–Fc decoy protein has demonstrated a beneficial effect on the development and progression of atherosclerotic plaques in mice. Furthermore, Fn14 deletion or anti-TWEAK administration diminished the volume of the ischemic lesion after stroke, a related complication of atherosclerotic plaque rupture. Although the use of monoclonal antibodies offers – unlike small-molecule drugs – high target specificity and allows less frequent, albeit parenteral, administration, there is a need for further drug development in this area, including Fn14-specific antagonists.

The role of the TWEAK/Fn14 axis on pathological vascular remodeling is not completely understood, and many questions need to be answered. Could TWEAK induce vascular remodeling in contexts other than atherosclerosis? As in stroke, could TWEAK participate in the outcome of myocardial infarction? Could anti-TWEAK therapy prevent atherosclerotic plaque development and progression in the presence of different cardiovascular risk factors such as diabetes or hypertension? Since TWEAK is also able to interact with CD163 (8), could overexpression of CD163 diminish the proatherogenic effect of TWEAK? Statins can diminish Fn14 expression in cultured SMCs (18), so could statins inhibit atherosclerotic plaque progression induced by TWEAK? In the near future, these and other potential questions could help us to understand the role of this axis in different cardiovascular pathologies.

Finally, the evidence accumulated indicates that sTWEAK could be a biomarker for the diagnosis and prognosis of CVD. The reduction of sTWEAK plasma levels has been demonstrated in subjects with different vascular affectations and its association with total and cardiovascular morbidity and mortality has been reported in different cohorts. However, after an acute cardiovascular event, sTWEAK concentrations are increased. Current and future studies in large-scale populations will help us to determine the relevance of sTWEAK as a CVD biomarker and its potential implementation in clinical practice.

## Conflict of Interest Statement

The author declares that the research was conducted in the absence of any commercial or financial relationships that could be construed as a potential conflict of interest.
